# Comparing the performance of single and multifrequency Kelvin probe force microscopy techniques in air and water

**DOI:** 10.3762/bjnano.13.82

**Published:** 2022-09-12

**Authors:** Jason I Kilpatrick, Emrullah Kargin, Brian J Rodriguez

**Affiliations:** 1 School of Physics and Conway Institute of Biomolecular and Biomedical Research, University College Dublin, Belfield, Dublin, D04 V1W8, Ireland

**Keywords:** AFM, atomic force microscopy, closed loop, Kelvin probe force microscope, KPFM, open loop, performance, signal-to-noise ratio

## Abstract

In this paper, we derive and present quantitative expressions governing the performance of single and multifrequency Kelvin probe force microscopy (KPFM) techniques in both air and water. Metrics such as minimum detectable contact potential difference, minimum required AC bias, and signal-to-noise ratio are compared and contrasted both off resonance and utilizing the first two eigenmodes of the cantilever. These comparisons allow the reader to quickly and quantitatively identify the parameters for the best performance for a given KPFM-based experiment in a given environment. Furthermore, we apply these performance metrics in the identification of KPFM-based modes that are most suitable for operation in liquid environments where bias application can lead to unwanted electrochemical reactions. We conclude that open-loop multifrequency KPFM modes operated with the first harmonic of the electrostatic response on the first eigenmode offer the best performance in liquid environments whilst needing the smallest AC bias for operation.

## Introduction

Atomic force microscopy (AFM) is an enabling technique for the nanoscale mapping of topography and surface properties of interfaces in a wide range of environments [1]. Kelvin probe force microscopy (KPFM) utilizes the application of a bias and a conductive probe to map the local electrical properties of an interface at the nanoscale [2], allowing for the determination of the local contact potential difference (CPD) between the probe and the sample. This, in turn, allows the work function of the sample to be measured if the work function of the probe is known and vice versa. The mapping of local electrical properties of the interface is essential to further our understanding of corrosion, sensing, solar cells, energy storage devices, and bioelectric interfaces [3–8]. Since its first application in 1991 [2], there have been significant developments in the field of KPFM [6,9–10] with significant advances in both temporal [11–14] and spatial resolution [13,15–19]. These advances have enabled investigations mapping light-induced surface potential dynamics [20], ferroelectric domains [19], individual quantum dots [21–22], and even submolecular charge distributions [23–27]. These applications demonstrate that KPFM is capable of atomic-scale spatial resolution and nanosecond time resolution under specific conditions.

KPFM-based techniques can largely be classified as either “open loop” (OL) or “closed loop” (CL). CL techniques employ a feedback loop to apply a bias to compensate for the electrostatic force (or force gradient) between the tip and sample. CL techniques are more common than OL techniques due to the ease of implementation, wide-scale availability, and direct measurement of the apparent CPD. OL techniques, by contrast, are feedback-free and can be used to determine the CPD without the need to apply a DC bias [28–29]. OL techniques are increasingly being adopted to enable the mapping of voltage-sensitive materials [30–32], to enable investigations of fast electrodynamic processes [11–14] and to enable measurements in liquid environments (where bias application could lead to stray currents and unwanted electrochemical reactions) [9,33–35]. OL techniques avoid the limitations and artefacts that can arise when using a feedback loop, for example, bandwidth limitations due to the time constant of the feedback loop [29], increased noise [36–37], and electrical crosstalk [38–39]. Whilst the application of DC bias is not required for OL operation it can still be utilized to allow CPD to be determined via bias sweeps [28,40] or to investigate gate-dependent potential profiles of interfaces [22,41–42]. There are a wide range of OL KPFM techniques beyond those examined in this paper, including pump-probe KPFM [13,20,43], time-resolved KPFM [11–12,44–47], fast free force recovery KPFM (G-Mode) [14,48–50], intermodulation electrostatic force microscopy (EFM) [42,51], and PeakForce tapping KPFM [52].

The fundamental detection sensitivity to electrostatic forces in KPFM is generally expressed as the minimum detectable CPD [53], 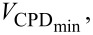 and is directly limited by the geometry of the interaction, thermal noise of the cantilever, and the detection noise limits of the AFM [36,54]. Cantilevers have a number of eigenmodes, ω*_n_*, where there is a mechanical enhancement in the response of the lever proportional to the quality factor of that mode, *Q_n_*, where *n* is the mode number [36,55]. KPFM techniques can be applied off resonance (ω ≠ ω*_n_*), where 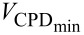 ∝ 1/*k_n_*, where *k_n_* is the spring constant of the *n*-th eigenmode. More generally, KPFM techniques are applied at, or close to ω*_n_* where 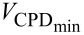 ∝ *Q_n_*/*k_n_* and there is a significant enhancement in the oscillation amplitude of the cantilever in response to the electrostatic force, thereby increasing the signal-to-noise ratio (SNR) [10]. In this paper we define the SNR as the ratio of the measured signal (oscillation amplitude of the cantilever) at a given frequency to the noise at that frequency. Furthermore, we use the conventional definition of 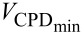 whereby SNR = 1 [2,53,56–59].

The desire to take advantage of the SNR enhancement on eigenmodes of the cantilever have led to the adoption of a number of imaging strategies [10]. The regulation of tip–sample distance in KPFM imaging is generally performed by employing a feedback loop that maintains the mechanical oscillation amplitude of the cantilever at the fundamental eigenmode, ω_1_, at a fixed value. This precludes simultaneous measurement of electrostatic forces on this eigenmode (the SNR is highest on ω_1_). As such, strategies for achieving high SNRs generally focus on three areas: (1) Lift mode – here the surface under investigation is mapped in two passes, the first pass with only a mechanical excitation applied at ω_1_ and the second pass with only the electrical excitation applied at ω_1_ as it traces the topography measured in the first pass at a specific lift height above the surface. Lift height can be set such that the electrostatic forces are isolated from stronger short range forces at the expense of spatial resolution [10,58]. By setting the lift height to match the mean tip–sample distance of the lever during the mechanical imaging pass, topography and potential can be correlated. (2) Sideband modes – here the electrical signal, ω_e_, is applied as a low frequency (ω_e_ ≪ ω_1_) such that the electrical and mechanical drive, ω_m_, form mixing products ω_m_ ± ω_e_. These mixing products have enhanced sensitivity to electrostatic forces at the expense of localization to small tip–sample separations. By choosing ω_e_ such that the mixing products fall on the sidebands of ω_1_, the SNR is improved whilst enabling single-pass scanning. There are trade-offs here in that ω_e_ should be higher than the topography feedback bandwidth to prevent crosstalk yet low enough that the mixing products are not too far from ω_m_ to take advantage of gains in the SNR. This limits the accessible bandwidth and, therefore, the scanning speed [10]. (3) Higher eigenmodes – by applying the electrical signal to a higher eigenmode, the electrostatic response can be measured simultaneously with topography in a single pass [17,60–62]. Higher eigenmodes typically have poorer SNRs than the fundamental eigenmode since *k_n_* increases more rapidly than *Q_n_* [60–61]. However, these modes still offer significant SNR enhancements over off-resonance techniques, higher spatial resolution due to reduced influence of the cantilever to the electrostatic forces [17,60,63], and higher bandwidth than side-band techniques [10,58]. All modes of KPFM can in principle be applied in any of these scenarios.

In addition, there are more exotic approaches to mapping using KPFM, for example, force volume [64], PeakForce tapping [19,52], and, although not yet reported, KPFM could be combined with the recently introduced photothermal off-resonance tapping (PORT) mode [65]. KPFM can also be combined with other techniques to yield multidimensional data sets and aid in isolation of the influence of electrostatic potential, for example, PeakForce infrared-KPFM (PFIR-KPFM) [66], nanomechanical mapping + KPFM [67–68], magnetic force microscopy (MFM) + KPFM [69], piezoresponse force microscopy (PFM) + KPFM [70], and G-mode [14,48–50].

The most common application of KPFM in AFM is CL AM-KPFM on the fundamental eigenmode where a bias feedback loop is employed to cancel the electrostatic force and to extract *V*_CPD_ [10,60–61]. This single-frequency technique can also be used under OL conditions without a feedback loop using phase-based detection [71], frequency sweeps [40,64,72], or bias modulation [10,52,73]. The advantages of CL AM-KPFM are that it is easy to implement, is standard on most commercial AFMs, and has high bias sensitivity [74]. The disadvantages of this technique are that it is limited by the properties of the feedback loop (and its associated artefacts) and is rarely fully quantitative due to the large influence of the cantilever beam on the electrostatic response [75–77]. This limits the spatial resolution. However, some authors address this by deconvolving the probe geometry from measurements in order to access a true surface potential map [78–79].

A natural extension of AM-KPFM is dual-harmonic KPFM (DH-KPFM), which is an OL technique that utilizes the measurement of both the first and second harmonic of the electrostatic response (ω_e_ and 2ω_e_). By combining these two components, *V*_CPD_ can be obtained directly without the need to employ a feedback loop, knowledge of the tip–sample capacitance gradient, or application of a DC bias. Initially implemented in ultrahigh vacuum by Takeuchi et al. [30], the method was extended to liquids by Kobayashi et al. [80] and to ambient environments by Collins et al. [81]. This technique exhibits a similar cantilever capacitive contribution to *V*_CPD_ as AM-KPFM and is only quantitative if the relative gain of the two measured frequencies is known either through an additional measurement or through modelling [29]. Since the electrostatic response occurs at ω_e_ and 2ω_e_, it is not possible to place both on eigenmodes in single-pass scanning, which adversely affects the SNR. To overcome this limitation, two passes could be made using excitation at ω_e_ and ω_e_/2 such that the required harmonics are measured at the same frequency. This approach is known as half-harmonic KPFM [82]. Alternatively, two electrical drives, ω_e1_ and ω_e2_, can be applied such that the required harmonics occur on eigenmodes. This allows for a direct OL access to *V*_CPD_ in a single pass with enhanced SNR. In addition, the mixing product, ω_mix_ = ω_e1_ ± ω_e2_ occurs and can be placed on an eigenmode in order to measure *V*_CPD_ with enhanced SNR since 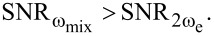 In this paper we refer to the use of two electrical drives and their products as electrodyne-KPFM (ED-KPFM).

In order to access higher spatial resolution, bandwidth, and SNR, heterodyne-KPFM (Het-KPFM) was developed [57] whereby the mechanical oscillation, ω_m_, at one eigenmode, used to track the topography, is mixed with ω_e_ such that ω_m_ ± ω_e_ occurs on another eigenmode [57]. Typically, the probe would be driven mechanically at ω_1_ and ω_e_ = ω_2_ − ω_1_ such that the first harmonic of the electrostatic response occurs at ω_2_. The positioning of the mechanical and electrical drives can also be applied such that the topography is measured on ω_2_ and the electrostatic response is on ω_1_ [58]. This allows for single-pass scanning with enhanced SNR with greater bandwidth than other KPFM techniques [10,58]. This technique combines the enhanced sensitivity from operating on eigenmodes with the enhanced spatial resolution due to the electrostatic response being proportional to the second derivative of the capacitance gradient, *C*″ [57,76,83–84]. This enhanced sensitivity to short range forces (up to three times more sensitive than frequency modulation KPFM [53]) removes the influence of the cantilever and delivers enhanced bandwidth due to the high frequency of ω_e_ [58]. Axt et al. found that Het-KPFM was the most accurate of all modes tested and was able to measure 99% of an applied potential difference even in the presence of strong stray fields [10]. Het-KPFM is generally operated in CL configurations [57–58] but could also be operated OL [58,85–86], either through bias sweeping techniques or through the simultaneous measurement of ω_m_ ± ω_e_ and ω_m_ ± 2ω_e_ similar to DH-KPFM. Het-KPFM has been demonstrated to achieve atomic resolution of the surface potential [53,76,86] and has enhanced our understanding of perovskite solar cells [10,87–88] and patch potentials in the Casimir force [89–90]. Implementations of Het-KPFM to date have primarily focused on the measurement of the first harmonic of the electrostatic force [57–58].

Furthermore, Het-KPFM can be extended using two electrical drive signals combined with a mechanical drive signal to aid in the positioning of the required harmonics on eigenmodes, enhancing both SNR and spatial resolution [91]. Examples include intermodulation AFM, which applies two electrical signals, ω_e1_ and ω_e2_, off resonance that mix with ω_m_ to generate sideband signals around ω_m_ [42,51], and harmonic mixing KPFM (HM-KPFM), which allows for the application of a combination of mechanical and electrical drives such that the first and second harmonic of the electrostatic response occurring at ω_m_ ± ω_e1_ and ω_m_ ± 2ω_e2_ both fall on an eigenmode, resulting in enhanced SNR [91]. Additionally, a ω_m_ ± ω_mix_ term occurs [83], which could also be placed on an eigenmode for enhanced SNR. The division of these electrostatic response components thereby enables the measurement of *V*_CPD_ in OL. Here, the signals may be coupled with the mechanical drive, which may be applied either at a higher eigenmode or off resonance, for example, in the PORT mode [65]. In this paper we refer to any application of two electrical signals with a mechanical drive as HM-KPFM.

For OL multifrequency KPFM techniques (DH, Het, ED, HM), there is a need to be able to determine the sign of *V*_CPD_ measured since we are dividing amplitude responses that are always positive. These techniques rely upon the sign of the cosine of the phase of the first harmonic electrostatic response [80]. However, for small *V*_CPD_ values, the phase is strongly affected by noise [42]. An alternative approach is to measure the phase difference between the electrostatic harmonics to enable the determination of the sign of the measured *V*_CPD_ [42]. In addition, the relationship between the response at different frequencies is strongly influenced by the transfer function of the cantilever. This frequency-dependent gain, XGain, represents the sensitivity ratio of the cantilever at the two frequencies of interest. XGain is relatively stable for a given tip–sample distance in a given environment and, as such, can be approximated mathematically [29]. However, changes in environment, tip–sample distance, and the influence of piezo-based mechanical activation significantly complicates these relationships and, as such, many techniques require the explicit measurement of XGain in order to be quantitative [29]. Spectral KPFM techniques (e.g., band excitation KPFM (BE-KPFM) [40,64,72], half-harmonic band excitation (HHBE-KPFM) [28,82], and G-mode [14,48–50]) that can measure the amplitude response of the cantilever as a function of frequency and DC bias, can access XGain directly as part of the measurements. Lastly, KPFM-based techniques can also utilize changes in XGain due to changes in the conservative [92–93] and dissipative [93–95] forces in order to access *V*_CPD_.

In order to assess the performance of OL and CL techniques and to establish the best route to obtain *V*_CPD_ in any environment with the smallest required bias, we directly compare AM, DH, Het, ED, and HM KPFM techniques in terms of the minimum detectable CPD, 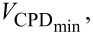 the minimum AC bias required for operation, 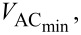 and the SNR. We compare and contrast the performance for three specific scenarios. The first scenario is “off resonance”, where the first harmonic of the electrostatic responses occurs on eigenmode ω_1_, and where the first harmonic of the electrostatic responses occurs on eigenmode ω_2_. We also compare the performance in air vs liquid (e.g., water), where both the transfer function of the cantilever changes (reducing *Q* enhancement at the eigenmodes) and the relative permittivity increases such that the electrostatic response is greatly enhanced. Other more complex effects in liquid environments are excluded from our analysis, for example, effects of the double layer, electrodynamics, or changes in permittivity with salt concentration. For a review of the impact of these effects on KPFM operation in liquid please see Collins et al. [9]. Our goal in this paper is to identify the OL techniques that provide the greatest performance with the smallest required *V*_AC_ for operation in liquid environments, where bias application could lead to stray currents and unwanted electrochemical reactions. Here, we restrict our analysis to the first two eigenmodes of a cantilever, where the SNR is highest, but these calculations could be extended to higher eigenmodes if desired [96].

## Performance Characteristics of KPFM Modes

In KPFM-based techniques an electrical bias is applied between a conductive AFM probe and a sample, Δ*U* = *V*_bias_ − *V*_CPD_, where *V*_bias_ may be a combination of DC bias, *V*_DC_, and an AC bias, *V*_AC_, applied at frequency ω_e_, for example, *V*_bias_ = *V*_DC_ + *V*_AC_ sin(ω_e_*t*). Here, *V*_bias_ may be applied either to the cantilever or to the sample. The application of Δ*U* results in an electrostatic force given by 
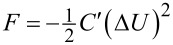
 where *C*′ is the capacitance gradient with respect to the tip–sample distance, *z*, and depends on the tip–sample geometry. By expanding the electrostatic force expression, we can obtain the amplitude response at DC, ω_e_ and 2ω_e_ (see Appendix II). In order to assess the performance of the various KPFM modes, we consider the conventional condition where the minimum detectable CPD, 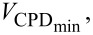 is defined as the conditions under which SNR = 1 [2,53,56–59]. For AM-KPFM we can solve the general equation for a single frequency response at ω_e_, where the noise due to the cantilever and AFM detection system, *N*(ω_e_), is equal to 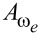, and obtain 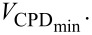 Similarly, we can rearrange this expression and obtain 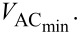 Here, we observe that both 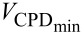 and 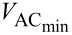 are proportional to *N*(ω_e_)/(*C*′*G*(ω_e_)). The SNR can also be obtained by taking the ratio between the amplitude response and the noise at ω_e_. For AM-KPFM operated under OL conditions (where *V*_DC_ = 0), SNR = 
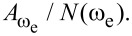
 These are the performance determining equations (see Table 1). Note that 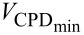 has the same expression for AM, DH, and ED modes and the performance determining factor is 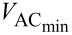, which depends on the relationship *N*(ω)/*G*(ω) at ω = 2ω_e_ for DH-based modes and ω = ω_mix_ for modes where two electrical drives are employed (ED). Mixing modes require √2 less *V*_AC_ than DH-based modes.

For heterodyne-KPFM both mechanical, ω_m_, and electrical, ω_e_, signals are applied. Under conditions where *V*_DC_ = 0 we can obtain the amplitudes of the mixing harmonics (see Appendix II) proportional to *A*_m_*C*″, where *A*_m_ is the mechanical oscillation amplitude of the cantilever and *C*″ is the derivative of the capacitance gradient with respect to *z*. Assuming *V*_AC_ = *V*_AC1_ = *V*_AC2_ we can repeat the procedure to obtain the performance determining equation for modes where electrical and mechanical signals are coupled. For these mechanically coupled modes the performance is proportional to *N*(ω_e_)/(*A*_m_*C*″*G*(ω_e_)) and again we find that modes that utilize a mixed electrical signal require √2 less *V*_AC_ than DH-based modes. Comparing purely electrical to mechanically coupled modes we observe that the former is more bias sensitive by 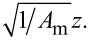


**Table 1:** Summary of performance determining equations.

Mode	Signal(s) measured	Amplitude	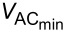	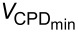

AM^a^	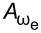	*C*’*V*_CPD_*V*_AC_*G*(ω_e_)	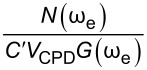	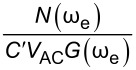
DH	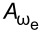	*C*’*V*_CPD_*V*_AC_*G*(ω_e_)	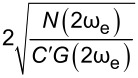	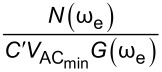
	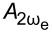	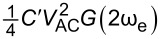		
ED-DH	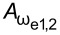	*C*’*V*_CPD_*V*_AC1,2_*G*(ω_e1,2_)	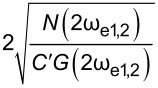	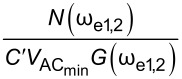
	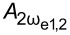	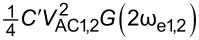		
ED-Mix	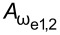	*C*’*V*_CPD_*V*_AC1,2_*G*(ω_e1,2_)	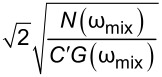	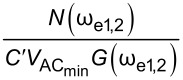
	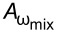	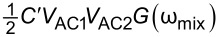		
Het^a^	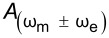	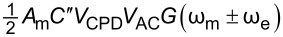	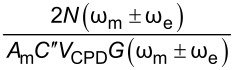	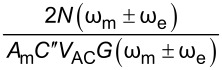
Het-DH	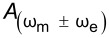	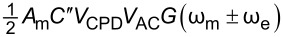	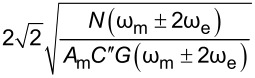	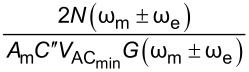
	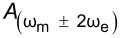	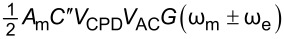		
HM-DH	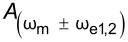	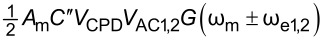	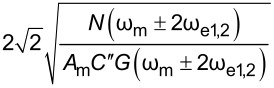	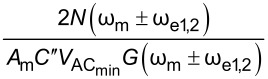
	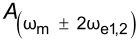	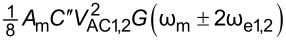		
HM-Mix	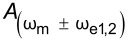	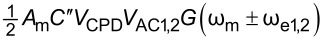	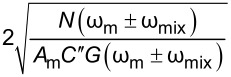	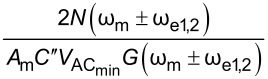
	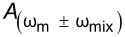	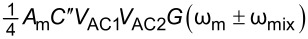		

^a^Designated single-frequency KPFM modes.

## Influence of Capacitance Gradient and Amplitude

**Figure 1:**
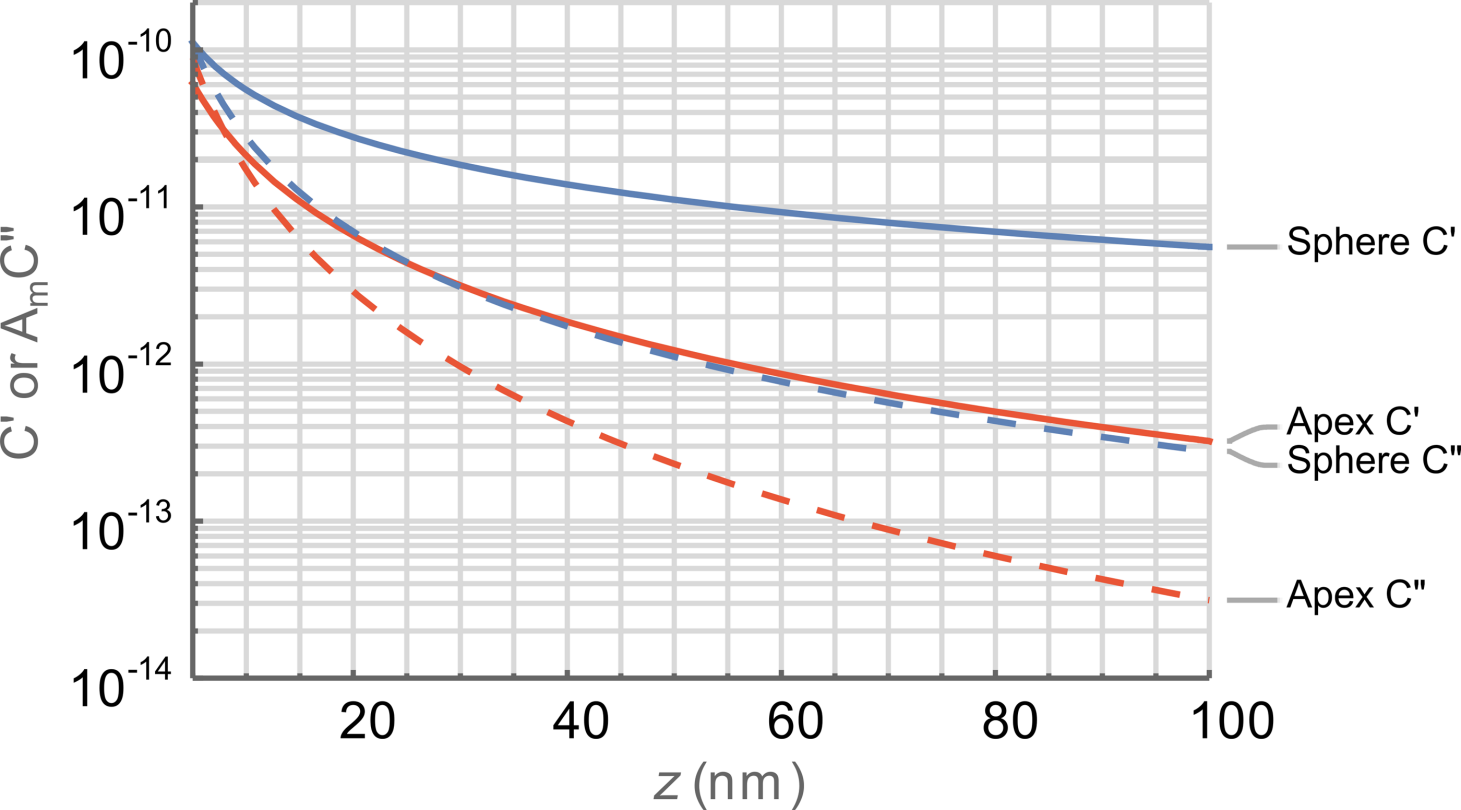
Comparison of apex and sphere capacitance functions as a function of the tip–sample separation, *z*. *C*′_Apex_ (red), *A*_m_*C*″_Apex_ (red dashed), *C*′_Sphere_ (blue), and *A*_m_*C*″_Sphere_ (blue dashed). Higher is better.

In this paper, we follow the approach originally employed by Nonnenmacher et al. [2] where the capacitance gradient is based on a sphere of radius *R* at a distance *z* from the surface such that


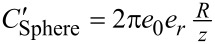


and


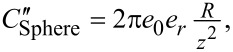


where *e*_0_ and *e*_r_ are the vacuum and relative permittivity, respectively. This approach is strictly only valid for *z* ≪ *R*. This simplistic expression only considers the capacitance contribution at the tip apex and ignores the overall geometry of the rest of the cantilever. The contribution at the tip apex is also commonly modelled as a spherical capped cone with [83,97]


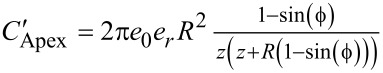


and


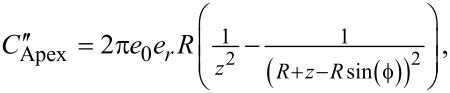


where ϕ is the half cone angle. Figure 1 shows the *z* dependence of the capacitance gradients using the sphere and cone models. Again, this simplistic expression only considers the capacitance contribution at the tip apex and ignores the overall geometry of the rest of the cantilever. For comparison we include *A*_m_*C*″ for both models as this is the equivalent term in the mechanically coupled KPFM modes. We observe that the capacitance gradient contribution of the end of the probe is greater for modes based on *C*′ (i.e., non-heterodyne modes) for both sphere- and apex-based models. This is helpful in understanding the observation that mechanically coupled modes require higher bias and have lower SNR than purely electrical modes.

We use simplified models of the tip–sample capacitance in this study to demonstrate the framework under which various KPFM modes can be directly compared. More complex models of the tip–cantilever system, which consider the additive contributions of the end of the tip (sphere or apex), the cone contribution, and the lever contribution (some including corrections for tilt of the cantilever with respect to the sample surface) are prevalent in the literature [16,98–102]. These more complex models are readily applicable to the performance determining equations in this paper as a simple substitution for *C*′ or *C*″. These more complex models will likely lead to a further widening in the performance differences between purely electrical and mechanically coupled KPFM-based modes as the *z* dependence is far stronger for *C*″. This increases spatial resolution at the cost of SNR and, thus, the required 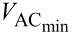.

Another important factor to consider in the operation of various KPFM modes is the influence of mechanical amplitude on performance. In CL operation the application of *V*_DC_ to nullify the present CPD results in the cantilever having zero amplitude when operated under ideal conditions [56]. By contrast OL modes rely on the detection of the amplitude of the cantilever at various frequencies. The amplitude of these oscillations will depend on both the magnitude of the signals (dependent upon *V*_AC_ and *V*_CPD_ and for the mechanically coupled modes on *A*_m_, see Appendix II) and the transfer function of the cantilever. Whilst there is an interest in obtaining large SNR values there is a trade-off in choosing an appropriate range for the mechanical amplitude of the cantilever. Under small amplitude conditions the tip can be positioned very close to the surface and as such there is an advantage in increasing the spatial resolution. The trade-off here is that the SNR will be small as the mechanical amplitude approaches the thermal noise limits of the cantilever. In addition, the positioning of the cantilever close to the surface will increase the damping on the cantilever and alter the transfer function accordingly [103]. Under conditions where the tip starts tapping the surface, the linearity of the amplitude signal in response to *V*_AC_ and/or *V*_CPD_ may be disrupted leading to artefacts in the measurements. For multifrequency techniques, this nonlinear motion of the cantilever due to tapping the surface can induce motion of the lever at higher harmonics and distort the frequency response characteristics of the system [104–106].

Under large amplitude conditions, the tip must be positioned further from the surface and as such there will be a reduction in SNR due to the need to integrate the signal over a larger distance. The larger tip–sample distance also means that more of the surface is sampled per oscillation and as such there will be a reduction in spatial resolution. The reader is directed to the Supporting Information of Garrett et al. [91] for a comprehensive discussion of the trade-offs of amplitude, resolution, and SNR.

## Results and Discussion

Here, we apply the equations established in Table 1 (and Appendix II) in order to compare and contrast the performance of KPFM techniques in both air and water environments. To do this, we examine three performance criteria, namely minimum detectable CPD, 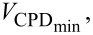 minimum required bias, 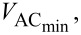 and SNR as a function of tip–sample separation, *z*, under the specified conditions as outlined in Table 2 in Appendix I. These metrics are examined for three cases: the first harmonic electrostatic response occurring off resonance, ω_off_, where ω_off_ ≪ ω_1_, on ω_1_, and on ω_2_. For modes that include a mechanical excitation at amplitude *A*_m_ of the lever, *z* is taken to be the mean tip–sample separation.

First, we compare single-frequency KPFM techniques (AM and Het) and observe that AM has advantages in terms of sensitivity with lower 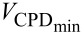 and a lower 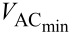 for all tip–sample separations for a given medium. The greater *z* dependence of the Het mode due to the dependence of *C*″ increases sensitivity at small separations, which leads to greater spatial resolution [53,58]. However, for a given medium the sensitivity never exceeds that of AM even under *z* = *A*_m_ conditions. We also observe the general trends associated with the change of medium in that operation in water results in lower values of 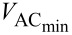 and 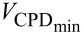 due to the increased relative permittivity of the medium.

**Figure 2:**
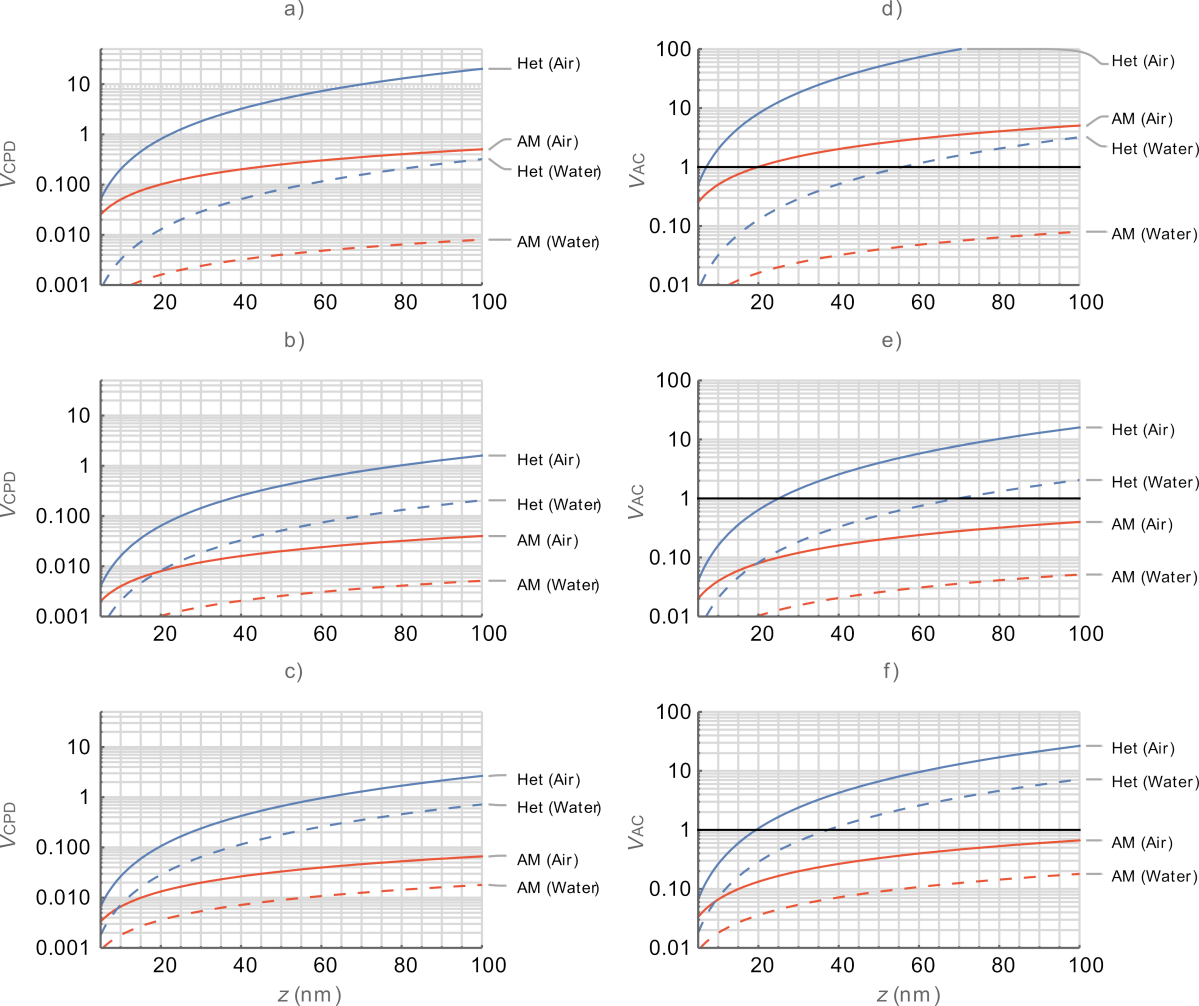
Performance characteristics of single-frequency detection techniques (AM and Het) as a function of the tip–sample separation, *z*. (a–c) 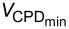 and (d–f) 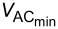 for the first harmonic electrostatic response occurring ω_off_ (top), ω_1_ (middle), and ω_2_ (bottom), respectively. AM (red) and Het (blue) for air (solid) and water (dashed). 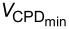 is calculated for *V*_AC_ = 1.0 V. 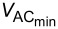 is calculated for *V*_CPD_ = 0.1 V. The black lines in (d–f) indicate *V*_AC_ = 1.0 V. Lower bias is better for all graphs.

**Figure 3:**
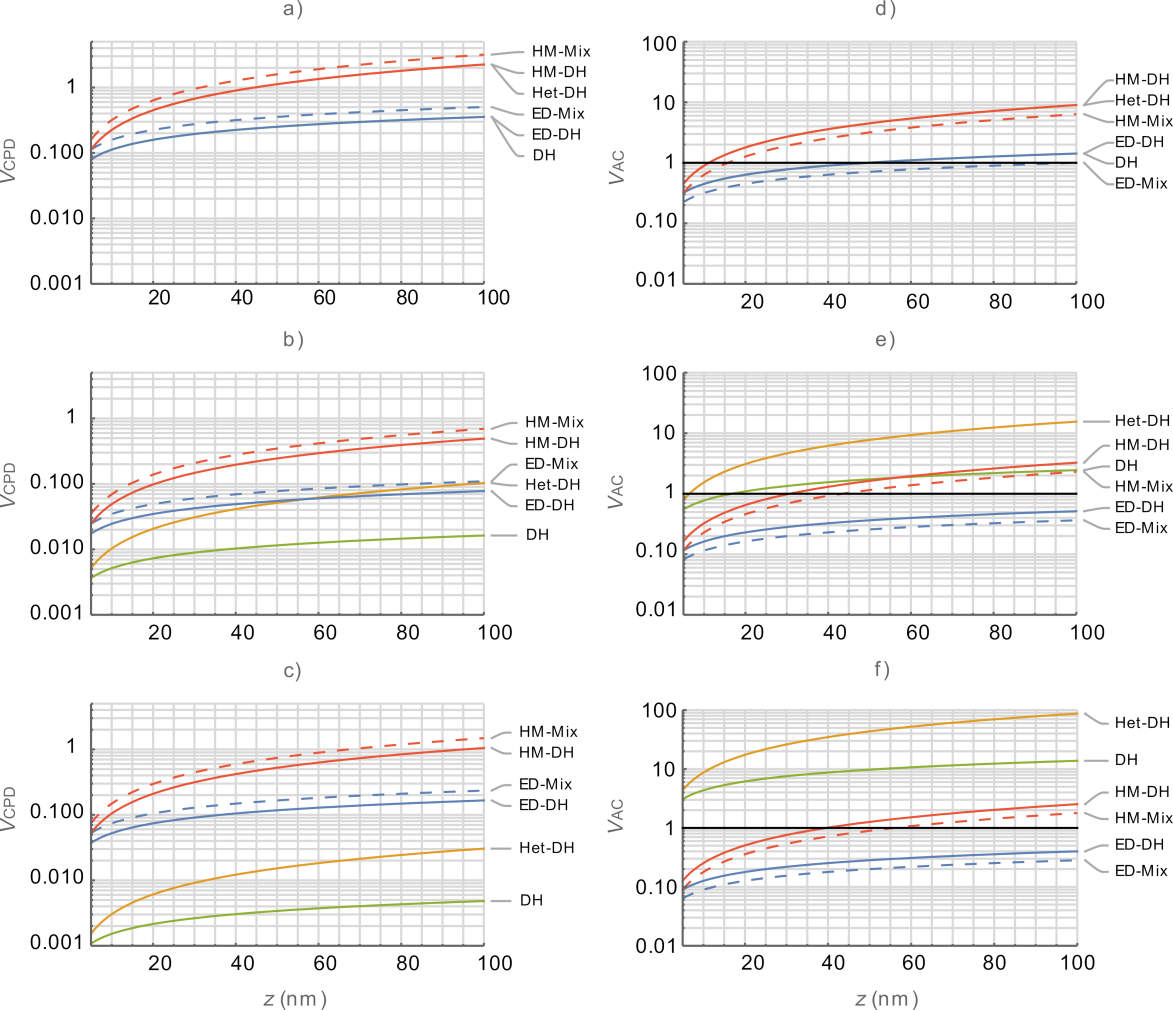
Comparison between multifrequency KPFM modes in air as a function of the tip–sample separation, *z*. (a–c) 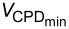 and (d–f) 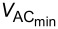 for the first harmonic electrostatic response occurring on ω_off_ (top), ω_1_ (middle), and ω_2_ (bottom), respectively. DH (green), Het-DH (orange), ED (blue), and HM (red). Mixed responses are shown with dashed lines. 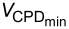 is calculated at *V*_AC_ = 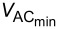 for all *z*. The black lines in (d–f) indicate *V*_AC_ = 1.0 V. Lower bias is better for all graphs.

**Figure 4:**
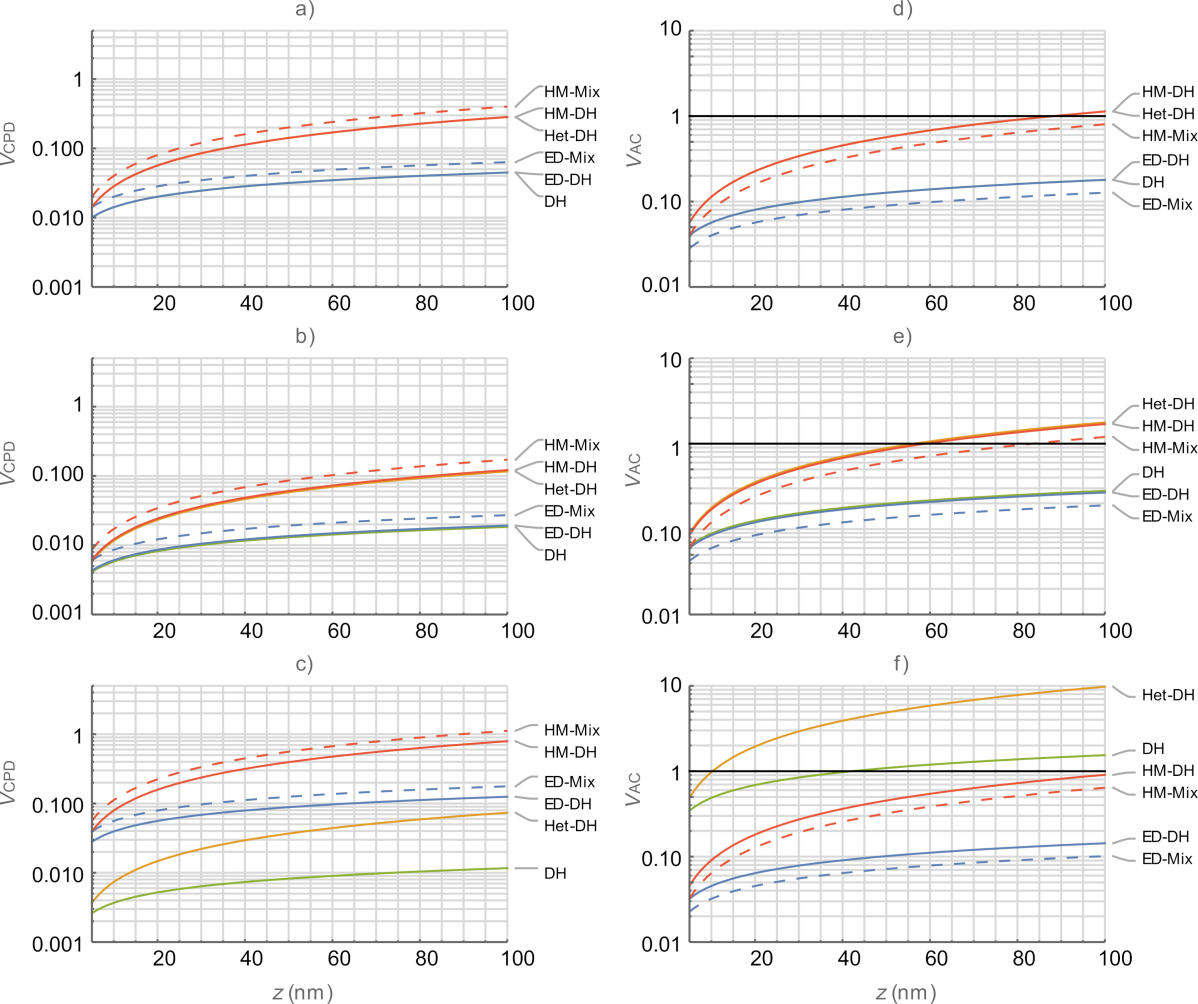
Comparison between OL KPFM modes in water as a function of the tip–sample separation, *z*. (a–c) 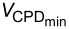 and (d–f) 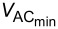 for the first harmonic electrostatic response occurring on ω_off_ (top), ω_1_ (middle), and ω_2_ (bottom), respectively. DH (green), Het-DH (orange), ED (blue), and HM (red). The black lines in (d–f) indicate 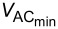 = 1.0 V. Mixed responses are shown with dashed lines. Lower bias is better for all graphs.

The solutions of the equations used in Figures 2–4 are under the conditions whereby SNR is strictly equal to 1 as defined in the formal definitions. Since this condition does not allow for practical operation, we impose an arbitrary minimum SNR of 10 (designated as *V*_AC_ = 1.0 V in Figures 2–4) and consider any performance below this criterion for a given set of conditions to be impractical to implement. As such, we observe that, for Figure 2 AM in air is impractical for ω_off_ for *z* > 20 nm whilst Het is impractical for conditions of *z* > 7 nm. This limitation is contrasted to operation on eigenmodes where AM is practical for both air and water at all separations presented. By contrast, Het is limited to operation at *z* < 70 nm under all conditions.

Figure 3 compares multifrequency KPFM techniques (DH, Het, ED, HM) in air and we observe that there is a general trend in 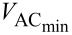 with mechanically coupled modes (Het and HM) requiring more bias than purely electrical modes (DH and ED). We also observed that responses based on Mix variants result in smaller 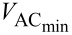 values and, consequently, slightly higher 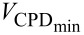 values for a given mode. For the ω_off_ case, we can observe a tight grouping of the mechanically coupled and purely electrical modes with the latter offering significantly better performance. Even with the reduced 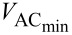 values of the purely electrical modes, 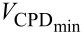 is still much lower for these modes. These trends continue to hold for operation on the eigenmodes. We observe that DH and Het-DH modes require significantly larger bias than other modes when operated on eigenmodes due to the poor placement of the second harmonic of the electrostatic response. This results in values for 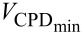 that appear better than those of other modes but are not practically achievable due to the high 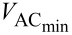 requirements. When both 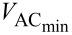 and 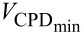 values are taken into consideration we observe that ED modes offer both the lowest 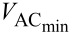 and the best corresponding 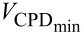 performance with practical operation below 0.5 V of AC bias and CPD resolution of less than 100 mV for the SNR = 1 condition on ω_1_. The performance of HM modes converges with that of the ED modes at the minimum tip–sample separation, where *z* = *A*_m_, but is significantly worse at large separations. The 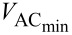 values for ED on ω_2_ are slightly lower than ω_1_ but this corresponds with poorer 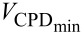 values. Whilst ED is practical at *z* > 100 nm on the eigenmodes, HM modes are limited to operation at *z* < 50 nm.

Figure 4 compares multifrequency KPFM techniques (DH, Het-DH, ED, and HM) under the same conditions as Figure 3 in a water medium. Here, both 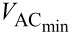 and 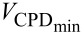 are reduced due to the increase in *e*_r_ from 1 to 80. Consequently, HM and ED modes can both be practically implemented both on and off the eigenmodes for *z* < 50 nm. A decision would need to be made between the higher spatial resolution afforded by HM-based modes and the increased sensitivity to CPD afforded by the ED modes. The grouping of performance into mechanically coupled and purely electrical modes is preserved for both ω_off_ and ω_1_ cases. This is due to the low *Q* and its negligible performance enhancement with only small differences between ω_off_ and ω_1_. We note that DH and Het-DH again suffer from poor frequency placement, however, this is only manifested when operating on ω_2_ where the higher *Q* has some impact.

**Figure 5:**
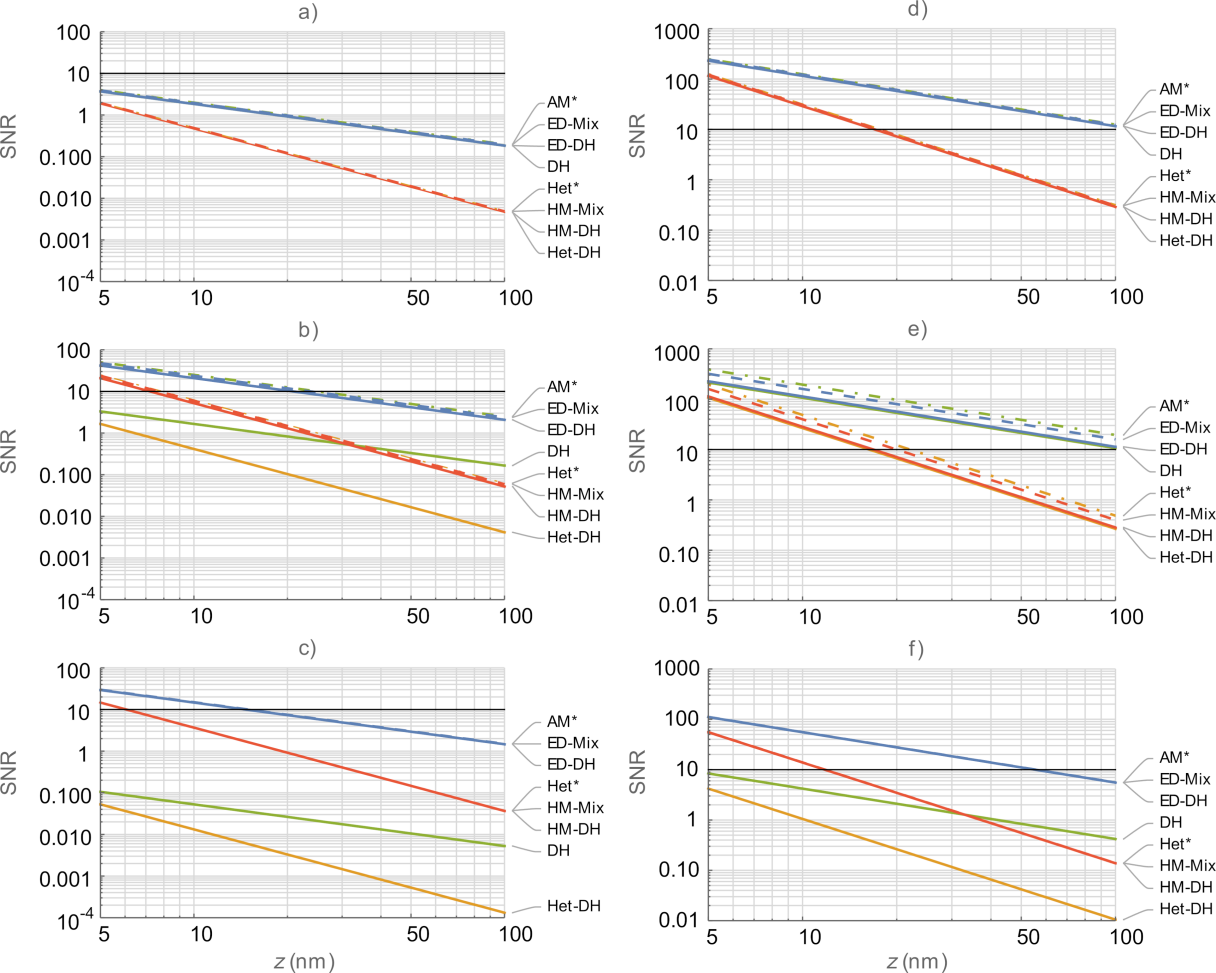
Comparison of SNR of KPFM modes in air (a–c) and water (d–f) for the first harmonic electrostatic response occurring on ω_off_ (top), ω_1_ (middle), and ω_2_ (bottom), respectively. DH (green), Het-DH (orange), ED (blue), and HM (red). Mixed responses are shown with dashed lines. Single frequency responses are shown with dash-dotted lines. *Designated single-frequency KPFM modes. The black lines in all graphs indicate SNR = 10. Higher SNR indicated better performance for all graphs.

Until now we have compared and contrasted the performance of single- and multifrequency KPFM modes by deriving the 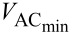 for SNR = 1 and then using these values to calculate the corresponding 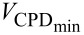 performance. In order to remove the interdependence of these two variables, we now examine the SNR-based performance of each mode under conditions where *V*_AC_ and *V*_CPD_ are standardized to values of 1.0 V and 0.1 V, respectively. Figure 5 shows the SNR calculations under these conditions for both air and water. For comparison, we include both the single-frequency and multifrequency KPFM modes. For air, we observe that no mode is capable of meeting the SNR = 10 performance criteria when operated at ω_off_. On ω_1_, AM-and ED-based modes are viable at *z* < 20 nm whilst Het- and HM-based modes are viable at *z* < 7 nm. The same modes are viable at smaller separations for ω_2_. DH and Het-DH modes are not viable for operation under any of the calculated conditions in air. For water, the increased relative permittivity increases the SNR of all modes. As such AM-, DH-, and ED-based modes are viable for ω_off_ for *z* < 100 nm and *z* < 18 nm for Het-, Het-DH-, and HM-based modes. This grouping remains largely unchanged when operated on ω_1_ with viability at *z* < 100 nm for the purely electrical modes and *z* < 20 nm for the mechanically coupled modes. The DH and Het-DH modes become unviable for ω_2_ and the remaining purely electrical modes are viable for *z* < 55 nm and the mechanically coupled modes are viable for *z* < 12 nm.

From this analysis, it is clear that under all of the conditions examined in this study the purely electrical modes offer a significant enhancement in terms of sensitivity when compared with the mechanically coupled analogues. Under conditions where implementation is required at larger tip–sample separations due to, for example, lift-mode, force volume mapping, surface topography limitations, or positioning of the tip with respect to double layer overlap, it is clear that the purely electrical modes offer significant performance enhancement over mechanically coupled modes. When implementation of KPFM at *z* ≤ *R* becomes feasible, the performance gap narrows significantly, and the technique choice will likely be based on ease of implementation and spatial resolution requirements of the experiment. For operation in liquid environments, ED-based modes offer very low *V*_AC_ requirements, optimal CPD detection, and high SNR. This mode is a clear choice under the conditions specified in this study with optimal performance observed when operating with the first harmonic of the electrostatic response on ω_1_ and the ω_mix_ signal placed on ω_2_. The use of probes with higher *Q_n_*/*k_n_* ratios would further enhance the performance of this mode. Choosing tips with larger end radius and working at a minimum tip–sample separation would also lower *V*_AC_ requirements, increase CPD detection limits, and increase SNR.

**Figure 6:**
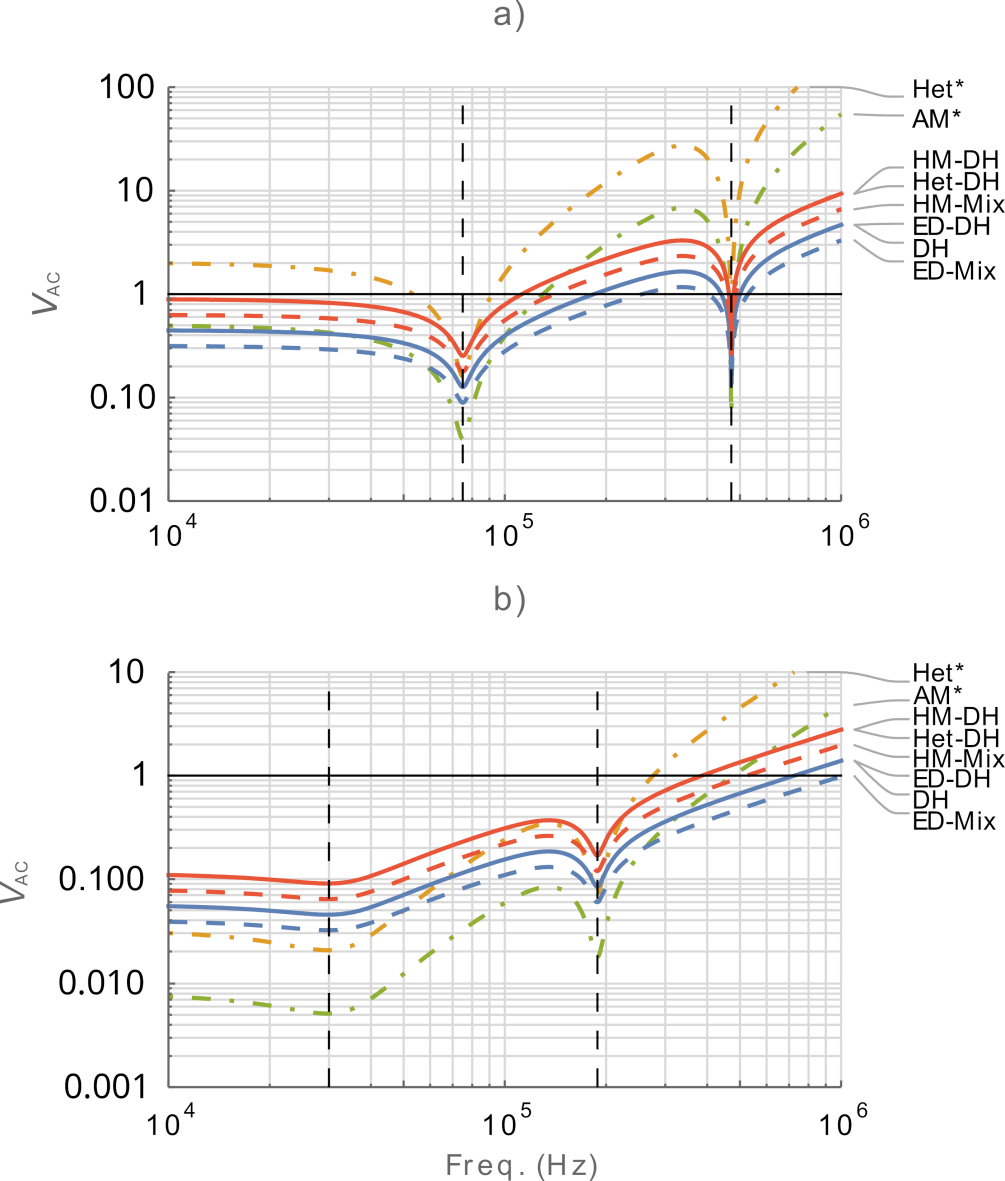
Comparison of the minimum AC bias required for operation, 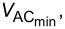 as a function of frequency in (a) air and (b) water. Calculations are made for *z* = 10 nm. The vertical dashed black lines indicate eigenmode positions. *Designated single-frequency modes. *V*_CPD_ = 0.1 V for single frequency modes. The solid black lines indicate 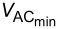 = 1.0 V. Lower values are better for all graphs.

Since the transfer function of the cantilever is fundamental in determining the performance of a given KPFM mode at a given frequency, it is important to understand its influence. Figure 6 shows the frequency dependence of 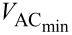 for air and water cases. Single-frequency modes are included for comparison. Note that previous calculations placed the drive frequencies such that the harmonics of the electrostatic response occurred in the desired locations with respect to the eigenmodes. For this figure, we calculate 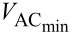 such that the relevant electrostatic response occurs at the frequency stated on the *x*-axis. This means we divide the input frequency for Equation 1 and Equation 2 by two for comparison. We observe that the minimum bias occurs on the eigenmodes with ω_1_ having a smaller 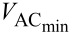 than ω_2_, as expected. The shape of the response for both air and water cases is proportional to *N*(ω)/*G*(ω). We also observe that there is a parallel relationship in 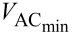 between the multifrequency modes with fixed ratios. The ratio between purely electrical and mechanically coupled modes is *A*_m_/2*z* whilst the ratio between modes with the suffix DH and Mix is √2. Single-frequency modes exhibit a stronger frequency dependence with AM having the lowest 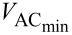 of any mode at the eigenmodes.

## Conclusion

In this work, we have presented the derivation of equations that allow the performance of a range of single- and multifrequency KPFM modes to be directly compared quantitatively. The relative performance of each mode is then presented in air and water in order to allow us to identify a suitable candidate for performing KPFM-based experiments in liquid with optimal response whilst minimizing the required low *V*_AC_ needed for operation in order to avoid the associated problems, for example, current flow, electrodeposition, or Faradaic reactions [9]. For this assessment, a number of simplifying assumptions have been made: (1) We use a simple spherical model of tip–sample capacitance [2,97] and do not consider the contributions from the rest of the cantilever. This would generally lead to an under-reporting of the superior voltage sensitivity (at the cost of lateral resolution) of the purely electrical modes in this work. (2) We consider both air and water as linear lossless dielectric media and do not consider additional properties such as electrodynamics or the effect of the tip–sample position relative to the position of the double layer. (3) We only compare frequency responses off resonance, ω_off_, and for the first two eigenmodes of the cantilever and combinations thereof. The driving frequencies are chosen such that the first harmonic of the electrostatic response signal will occur either on ω_off_ (ω_e_ ≪ ω_1_), or on ω_1_ or ω_2_. In practice, KPFM can be performed at any chosen frequency. (4) For modes including a mechanical excitation of the lever, the response is calculated at the mean tip–sample separation. The true instantaneous response would depend on the tip–sample separation during the oscillation cycle. (5) We consider the capacitive terms to be equivalent for different eigenmodes, allowing for a direct division of the electrostatic force harmonics in order to access CPD. Some authors have indicated that the capacitance contribution of the lever differs at higher eigenmodes due to the mode shape of the cantilever [17,60,63]. This would primarily affect the accuracy of CPD for purely electrical modes dependent on *C*′ as the mechanically coupled modes dependent on *C*″ have minimal cantilever contribution [101]. Any difference in capacitance (and therefore corrections that need to be applied) could be established by measuring the *z* dependence of the second harmonic of the electrostatic force for each eigenmode [29,83,98].

Whilst these simplifications are applied in this paper, the equations presented herein can be extended and applied to experiments with increased complexity. As such, we consider these equations to serve as the basis for planning KPFM-based experiments and assessing relative performance of KPFM-based modes. Furthermore, Table 3 in Appendix III provides a comprehensive guide to the various drive and detection schemes that are necessary to implement each of the KPFM modes presented in this paper along with a brief description of the advantages and disadvantages of each technique.

Clear trends are present in the comparison of the modes throughout: (1) Purely electrical modes require smaller *V*_AC_ and have better CPD detection and SNR than their mechanically coupled analogues. Each of these performance metrics is decreased by a factor of *A*_m_/2*z* for the mechanically coupled analogues of the purely electrical modes. (2) DH modes suffer significantly from problems with frequency placement relative to the transfer function of the cantilever. This could be remedied by operation of these modes using a half-harmonic [40,82] approach, which would greatly improve performance and remove reliance of XGain at the expense of having to scan the sample twice (once for each harmonic of the electrostatic response) and the associated errors due to sample drift or other temporal changes in the state of the tip and/or the sample. (3) Performance is optimal when the first harmonic of the electrostatic force occurs on the first eigenmode. (4) Smaller *V*_AC_ is required when operating in liquid environments due to the increased relative permittivity. We note that in the presence of ions there will be a strong *z* dependence, which may restrict practical operation to *z* ≤ *R* [107]. (5) Modes reliant upon Mix terms are superior in performance to those based on DH terms by a factor of √2.

As the transfer function of the cantilever significantly influences KPFM performance and multifrequency OL KPFM techniques become more popular for applications in liquid environments, the choice of cantilever geometry is becoming an increasingly important factor to consider in planning experiments and optimizing performance. Through mechanical design of the cantilever the placement of eigenmodes and their relative *Q_n_*/*k_n_* ratios can be optimized for increased performance of a given KPFM mode [108–110].

Whilst single-frequency KPFM modes are the most ubiquitous and easy to implement, their typical operation requires the application of a DC bias, which makes them unsuitable for applications in liquid environments. Even when these modes can be operated in an OL manner, they require explicit knowledge of *C*′ or *C*″, either through measurement or modeling, which can be difficult to establish in liquids in the presence of ions. Multifrequency OL KPFM-based approaches are therefore preferred in liquid environments. The ability to place multiple harmonics on eigenmodes to enhance performance in a single pass is preferable over multi-pass DH-based techniques. As such, ED and HM are the leading candidates for liquid operation. ED mixed modes with low bias requirements are a superior choice for larger (*z* > *R*) tip–sample separations whereas operation close to the surface is comparable to HM. For this case, the choice relies on the need for increased spatial resolution, which HM provides despite the dependency on *C*″. The use of Mix modes, where there is a contribution from both of the applied electrical drive signals, offers superior performance over DH-based modes of operation. As such, we conclude that ED-Mix and HM-Mix are the ideal candidates for KPFM-based measurements in liquid environments.

## Appendix I: Calculation Parameters

For calculation parameters see Table 2.

**Table 2:** Parameters used for calculations (air and water).

Constants	Value air (water)	Cantilever properties	Value air (water)

*k*_B_	1.38064852 × 10^−23^ J/K	*f*_1_	75,000 (30,000) Hz
*e*_0_	8.8541878 × 10^−12^ F/m	ω_1_	2π *f*_1_ Hz
*e*_r_	1 (80)	ω_2_	4π^2^ *f*_1_ Hz
*T*	300 K	*Q*_1_	200 (5)
*N*_d_	100 × 10^−15^ m/ 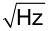	*Q*_2_	600 (15)
*B*	100 Hz	*k*_1_	2.8 N/m
*V*_AC_	1.0 V	*k*_2_	110 N/m
*V*_CPD_	0.1 V	*A*_m_	5 nm
		*R*	10 nm

## Appendix II: Derivation of Performance Determining Equations

The noise of a cantilever under ambient conditions at frequency ω, in a given bandwidth *B*, is defined as


[3]

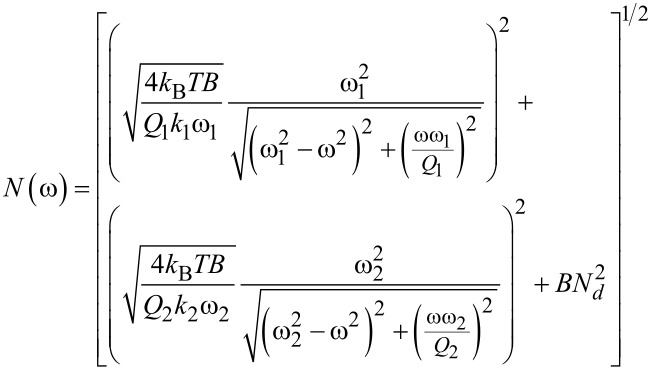



for the first two eigenmodes, where *k*_B_ is Boltzmann's constant, *T* is the temperature, *N*_d_ is the detector noise, and ω*_n_*, *k_n_*, and *Q_n_* are, respectively, the resonance frequency, spring constant, and quality factor of the *n*-th eigenmode (*n* = 1, 2). The corresponding gain at a given frequency is then defined as


[4]

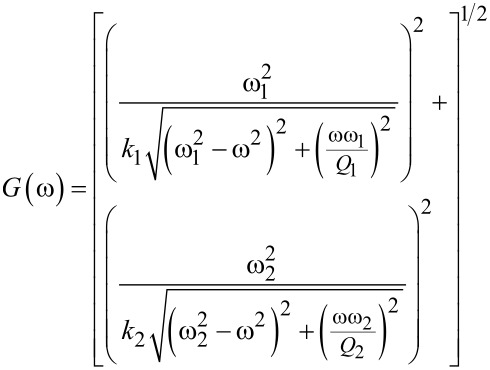



We note that more complex expressions for the transfer function of the cantilever are present in the literature and that these models may better represent the behavior of the lever in different environments [111–116].

## A. Amplitude Modulation KPFM (AM)

In KPFM-based techniques, a bias is applied between a conductive AFM probe and a sample


[5] $$\Delta U=V_{\text{DC}}+V_{\text{AC}}\sin\left(\omega_{e}t\right)-V_{\text{CPD}}$$

where *V*_DC_ is the DC bias and *V*_AC_ is the AC bias applied at frequency ω_e_. Here, the bias may be applied either to the cantilever or to the sample. The application of Δ*U* results in an electrostatic force given by


[6] $$F=-\frac{1}{2}C^{\prime }{\left(\Delta U\right)}^{2}$$

where *C*′ is the capacitance gradient with respect to tip–sample distance, *z*, and ends on the tip–sample geometry (see section "Influence of Capacitance Gradient and Amplitude"). By combining Equation 5 and Equation 6 we can obtain force components at DC, ω_e_ and 2ω_e_ (assuming *V*_DC_ = 0).


[7] $$\left|F_{\text{DC}}\right|=\frac{1}{2}C^{\prime }\left(V_{\text{CPD}}^{2}+\frac{1}{2}V_{\text{AC}}^{2}\right)$$

[8] $$\left|F_{\omega_{\text{e}}}\right|=C^{\prime }V_{\text{CPD}}V_{\text{AC}}\sin\left(\omega_{\text{e}}t\right)$$

[9] $$\left|F_{2\omega_{\text{e}}}\right|=\frac{1}{4}C^{\prime }V_{\text{AC}}^{2}\cos\left(2\omega_{\text{e}}t\right)$$

Thus, the amplitude responses at ω_e_ and 2ω_e_ are given as


[10] $$A_{\omega_{\text{e}}}=C^{\prime }V_{\text{CPD}}V_{\text{AC}}G\left(\omega_{\text{e}}\right)$$

[11] $$A_{2\omega_{\text{e}}}=\frac{1}{4}C^{\prime }V_{\text{AC}}^{2}G\left(2\omega_{\text{e}}\right)$$

In order to assess the performance of the various KPFM modes, we consider the conventional condition where the minimum detectable CPD, 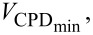 is defined as the condition under which SNR = 1 [2,53,56–59]. For AM-KPFM we can solve the general equation for a single-frequency response at ω_e_


[12] $$N\left(\omega_{\text{e}}\right)=A_{\omega_{\text{e}}}$$

for *V*_CPD_. By substituting Equation 10 into Equation 12 we obtain


[13] $$V_{{\text{CPD}}_{\min\left(\text{AM}\right)}}=\frac{N\left(\omega_{\text{e}}\right)}{C^{\prime }V_{\text{AC}}G\left(\omega_{\text{e}}\right)}$$

Similarly, we can rearrange Equation 13 for 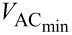 and obtain


[14] $$V_{{\text{AC}}_{\min\left(\text{AM}\right)}}=\frac{N\left(\omega_{\text{e}}\right)}{C^{\prime }V_{\text{CPD}}G\left(\omega_{\text{e}}\right)}$$

The SNR is then defined as


[15] $${\text{SNR}}_{\text{AM}}=\frac{A_{\omega_{\text{e}}}}{N\left(\omega_{\text{e}}\right)}$$

By substituting Equation 10 into Equation 15 we obtain


[16] $${\text{SNR}}_{\text{AM}}=\frac{C^{\prime }V_{\text{CPD}}V_{\text{AC}}G\left(\omega_{\text{e}}\right)}{N\left(\omega_{\text{e}}\right)}$$

Here, the SNR is specific to OL operation as in CL the feedback loop will reduce 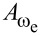 through the application of a DC bias such that 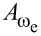 = *N*(ω_e_) (assuming ideal feedback and no instrument artefacts). Under these conditions, the SNR would always be 1 for CL operation.

## B. Dual Harmonic KPFM (DH)

In DH-KPFM the amplitude responses at 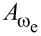 and 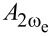 are measured and *V*_CPD(DH)_ is determined as [80]


[17] $$V_{\text{CPD(DH)}}=s\frac{A_{\omega_{\text{e}}}}{A_{2\omega_{\text{e}}}}\frac{V_{\text{AC}}}{4\text{XGain}\left(\omega_{\text{e}},2\omega_{\text{e}}\right)}$$

where s = 
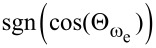
 (where 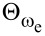 is the phase of the first harmonic electrical response) determines the sign of *V*_CPD_, and


[18] $$\text{XGain}\left(\omega_{\text{e}},2\omega_{\text{e}}\right)=\frac{G\left(\omega_{\text{e}}\right)}{G\left(2\omega_{\text{e}}\right)}$$

Whilst the 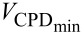 for DH-KPFM is the same as Equation 13 for the first harmonic response, we are primarily interested in the performance of the techniques as a function of 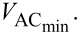 Here, 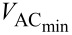 is determined from the bias required to satisfy the SNR = 1 condition for 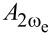 whereby


[1] $$V_{{\text{AC}}_{\min\left(\text{DH}\right)}}=2\sqrt{\frac{N\left(2\omega_{\text{e}}\right)}{C^{\prime }G\left(2\omega_{\text{e}}\right)}}$$

Substituting Equation 1 into Equation 13 we obtain 
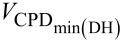
 under conditions where 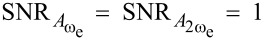 and


[19] $$V_{{\text{CPD}}_{\min\left(\text{DH}\right)}}=\frac{N\left(\omega_{\text{e}}\right)}{2C^{\prime }G\left(\omega_{\text{e}}\right)\sqrt{\frac{N\left(2\omega_{\text{e}}\right)}{C^{\prime }G\left(2\omega_{\text{e}}\right)}}}$$

SNR is then obtained from the root sum squared of 
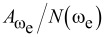
 and 
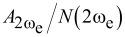
 resulting in


[20] $${\text{SNR}}_{\text{DH}}={\left(\begin{array}{l} {\left(\frac{N\left(\omega_{\text{e}}\right)}{C^{\prime }V_{\text{CPD}}V_{\text{AC}}G\left(\omega_{\text{e}}\right)}\right)}^{2}+\\ {\left(\frac{4N\left(2\omega_{\text{e}}\right)}{C^{\prime }V_{\text{AC}}^{2}G\left(2\omega_{\text{e}}\right)}\right)}^{2} \end{array}\right)}^{-1/2}$$

## C. Heterodyne-KPFM (Het)

For heterodyne-KPFM, both mechanical, ω_m_, and electrical, ω_e_, signals are applied. Under conditions where *V*_DC_ = 0 (typical of OL operation) we obtain


[21] $$\left|F_{\omega_{\text{e}}}\right|=C^{\prime }V_{\text{CPD}}V_{\text{AC}}\sin\left(\omega_{\text{e}}t\right)$$

[22] $$\left|F_{2\omega_{\text{e}}}\right|=\frac{1}{4}C^{\prime }V_{\text{AC}}^{2}\cos\left(2\omega_{\text{e}}t\right)$$

[23] $$\left|F_{\left(\omega_{\text{m}}\pm \omega_{\text{e}}\right)}\right|=\frac{1}{2}A_{\text{m}}C^{\prime \prime }V_{\text{CPD}}V_{\text{AC}}\sin\left(\left(\omega_{\text{m}}\pm \omega_{\text{e}}\right)t\right)$$

[24] $$\left|F_{\left(\omega_{\text{m}}\pm 2\omega_{\text{e}}\right)}\right|=\frac{1}{8}A_{\text{m}}C^{\prime \prime }V_{\text{AC}}^{2}\cos\left(\left(\omega_{\text{m}}\pm 2\omega_{\text{e}}\right)t\right)$$

where *C*″ is the derivative of the capacitance gradient with respect to *z*. Thus, the amplitudes are given by


[25] $$A_{\omega_{\text{e}}}=C^{\prime }V_{\text{CPD}}V_{\text{AC}}G\left(\omega_{\text{e}}\right)$$

[26] $$A_{2\omega_{\text{e}}}=\frac{1}{4}C^{\prime }V_{\text{AC}}^{2}G\left(2\omega_{\text{e}}\right)$$

[27] $$A_{\left(\omega_{\text{m}}\pm \omega_{\text{e}}\right)}=\frac{1}{2}A_{\text{m}}C^{\prime \prime }V_{\text{CPD}}V_{\text{AC}}G\left(\omega_{\text{m}}\pm \omega_{\text{e}}\right)$$

[28] $$A_{\left(\omega_{\text{m}}\pm 2\omega_{\text{e}}\right)}=\frac{1}{8}A_{\text{m}}C^{\prime \prime }V_{\text{AC}}^{2}G\left(\omega_{\text{m}}\pm 2\omega_{\text{e}}\right)$$

Generally, Het-KPFM is operated in CL employing a feedback loop applying a DC bias to the system to nullify the response at 
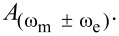
 Alternatively, knowledge of the capacitance gradient and transfer function of the cantilever would allow the CPD to be determined without employing a feedback loop. For Het-KPFM where only 
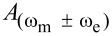
 is measured


[29] $$V_{{\text{CPD}}_{\min\left(\text{Het}\right)}}=\frac{2N\left(\omega_{\text{m}}\pm \omega_{\text{e}}\right)}{A_{\text{m}}C^{\prime \prime }V_{\text{AC}}G\left(\omega_{\text{m}}\pm \omega_{\text{e}}\right)}$$

where


[30] $$V_{{\text{AC}}_{\min\left(\text{Het}\right)}}=\frac{2N\left(\omega_{\text{m}}\pm \omega_{\text{e}}\right)}{A_{\text{m}}C^{\prime \prime }V_{\text{CPD}}G\left(\omega_{\text{m}}\pm \omega_{\text{e}}\right)}$$

*V*_CPD_ can also be obtained for OL operation of Het-KPFM by dividing Equation 27 by Equation 28 (analogous to DH-KPFM) to obtain


[31] $$\begin{array}{c} V_{\text{CPD(Het-DH)}}=s\frac{A_{\left(\omega_{\text{m}}\;\pm \;\omega_{\text{e}}\right)}}{A_{\left(\omega_{\text{m}}\;\pm \;2\omega_{\text{e}}\right)}}\cdot \\ \frac{V_{\text{AC}}}{4\text{XGain}\left(\omega_{\text{m}}\pm \omega_{\text{e}},\omega_{\text{m}}\pm 2\omega_{\text{e}}\right)} \end{array}$$

where


[2] $$V_{{\text{AC}}_{\min\left(\text{Het-DH}\right)}}=2\sqrt{2}\sqrt{\frac{N\left(\omega_{\text{m}}\pm 2\omega_{\text{e}}\right)}{A_{\text{m}}C^{\prime \prime }G\left(\omega_{\text{m}}\pm 2\omega_{\text{e}}\right)}}$$

Thus, measuring signals at ω_m_ ± ω_e_ and ω_m_ ± 2ω_e_ is required. Substituting Equation 2 into Equation 29 we obtain


[32] $$\begin{array}{c} V_{{\text{CPD}}_{\min\left(\text{Het-DH}\right)}}=\frac{N\left(\omega_{\text{m}}\pm \omega_{\text{e}}\right)}{\sqrt{2}A_{\text{m}}C^{\prime \prime }G\left(\omega_{\text{m}}\pm \omega_{\text{e}}\right)}\cdot \\ \frac{1}{\sqrt{\frac{N\left(\omega_{\text{m}}\pm 2\omega_{\text{e}}\right)}{A_{\text{m}}C^{\prime \prime }G\left(\omega_{\text{m}}\pm 2\omega_{\text{e}}\right)}}} \end{array}$$

The SNR is then obtained from the root sum squared of 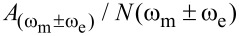 and 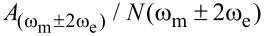 resulting in


[33] $${\text{SNR}}_{\text{Het-DH}}={\left(\begin{array}{l} {\left(\frac{2N\left(\omega_{\text{m}}\pm \omega_{\text{e}}\right)}{A_{\text{m}}C^{\prime \prime }V_{\text{CPD}}V_{\text{AC}}G\left(\omega_{\text{m}}\pm \omega_{\text{e}}\right)}\right)}^{2}+\\ {\left(\frac{8N\left(\omega_{\text{m}}\pm 2\omega_{\text{e}}\right)}{A_{\text{m}}C^{\prime \prime }V_{\text{AC}}^{2}G\left(\omega_{\text{m}}\pm 2\omega_{\text{e}}\right)}\right)}^{2} \end{array}\right)}^{-1/2}$$

## D. Electrodyne-KPFM (ED)

For electrodyne-KPFM, two electrical signals, ω_e1_ and ω_e2_, are applied and these generate an array of mixing products. Here,


[34] $$\Delta U=V_{\text{AC1}}\sin\left(\omega_{\text{e1}}t\right)+V_{\text{AC2}}\sin\left(\omega_{\text{e2}}t\right)-V_{\text{CPD}}$$

Substituting Equation 34 into Equation 6 we obtain [51,83]


[35] $$\left|F_{\text{DC}}\right|=\frac{1}{2}C^{\prime }\left(V_{\text{CPD}}^{2}+\frac{1}{2}V_{\text{AC1}}^{2}+\frac{1}{2}V_{\text{AC2}}^{2}\right)$$

[36] $$\left|F_{\omega_{\text{e}1,2}}\right|=C^{\prime }V_{\text{CPD}}V_{\text{AC}1,2}\sin\left(\omega_{\text{e}1,2}t\right)$$

[37] $$\left|F_{2\omega_{\text{e}1,2}}\right|=\frac{1}{4}C^{\prime }V_{\text{AC}1,2}^{2}\cos\left(2\omega_{\text{e}1,2}t\right)$$

[38] $$\left|F_{\omega_{\text{mix}}}\right|=\frac{1}{2}C^{\prime }V_{\text{AC}1}V_{\text{AC}2}\left(\begin{array}{l} \cos\left(\left(\omega_{\text{e}1}-\omega_{\text{e}2}\right)t\right)-\\ \cos\left(\left(\omega_{\text{e}1}-\omega_{\text{e}2}\right)t\right) \end{array}\right)$$

where ω_e1_ > ω_e2_. The amplitude responses are thus


[39] $$A_{\omega_{\text{e}1,2}}=C^{\prime }V_{\text{CPD}}V_{\text{AC}1,2}G\left(\omega_{\text{e}1,2}\right)$$

[40] $$A_{2\omega_{\text{e}1,2}}=\frac{1}{4}C^{\prime }V_{\text{AC}1,2}^{2}G\left(2\omega_{\text{e}1,2}\right)$$

[41] $$A_{\omega_{\text{mix}}}=\frac{1}{2}C^{\prime }V_{\text{AC}1}V_{\text{AC}2}G\left(\omega_{\text{mix}}\right)$$

where ω_mix_ = ω_e1_ ± ω_e2_. Here, we will assume *V*_AC_ = *V*_AC1_ = *V*_AC2_. Thus, *V*_CPD(ED-DH)_ can be obtained by combinations of 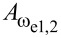 and 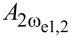 in a similar manner to DH-KPFM [51] where


[42] $$V_{\text{CPD(ED-DH)}}=s\frac{A_{\omega_{\text{e}1,2}}}{A_{2\omega_{\text{e}1,2}}}\frac{V_{\text{AC}}}{4\text{XGain}\left(\omega_{\text{e}1,2},2\omega_{\text{e}1,2}\right)}$$

or by combining 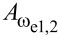 with 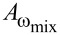 such that


[43] $$V_{\text{CPD(ED-Mix)}}=s\frac{A_{\omega_{\text{e}1,2}}}{A_{\omega_{\text{mix}}}}\frac{V_{\text{AC}}}{2\text{XGain}\left(\omega_{\text{e}1,2},\omega_{\text{mix}}\right)}$$

We can then determine the 
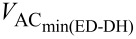
 for 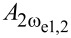 such that


[44] $$V_{{\text{AC}}_{\text{min(ED-DH)}}}=2\sqrt{\frac{N\left(2\omega_{\text{e}1,2}\right)}{C^{\prime }G\left(2\omega_{\text{e}1,2}\right)}}$$

and thus


[45] $$V_{{\text{CPD}}_{\text{min(ED-DH)}}}=\frac{N\left(\omega_{\text{e}1,2}\right)}{2C^{\prime }G\left(\omega_{\text{e}1,2}\right)\sqrt{\frac{N\left(2\omega_{\text{e}1,2}\right)}{C^{\prime }G\left(2\omega_{\text{e}1,2}\right)}}}$$

Similarly, for the case of 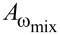 we can obtain


[46] $$V_{{\text{AC}}_{\text{min(ED-Mix)}}}=\sqrt{2}\sqrt{\frac{N\left(\omega_{\text{mix}}\right)}{C^{\prime }G\left(\omega_{\text{mix}}\right)}}$$

which results in


[47] $$V_{{\text{CPD}}_{\text{min(ED-Mix)}}}=\frac{N\left(\omega_{\text{e}1,2}\right)}{\sqrt{2}C^{\prime }G\left(\omega_{\text{e}1,2}\right)\sqrt{\frac{N\left(\omega_{\text{mix}}\right)}{C^{\prime }G\left(\omega_{\text{mix}}\right)}}}$$

and the resulting SNR is then obtained from the root sum squared of 
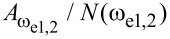
 and 
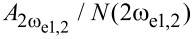
 resulting in


[48] $${\text{SNR}}_{\text{ED-DH}}={\left(\begin{array}{l} {\left(\frac{N\left(\omega_{\text{e}1,2}\right)}{C^{\prime }V_{\text{CPD}}V_{\text{AC}}G\left(\omega_{\text{e}1,2}\right)}\right)}^{2}+\\ {\left(\frac{4N\left(2\omega_{\text{e}1,2}\right)}{C^{\prime }V_{\text{AC}}^{2}G\left(2\omega_{\text{e}1,2}\right)}\right)}^{2} \end{array}\right)}^{-1/2}$$

Similarly, using the root sum squared of 
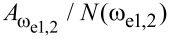
 and 
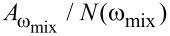
 we obtain


[49] $${\text{SNR}}_{\text{ED-Mix}}={\left(\begin{array}{l} {\left(\frac{N\left(\omega_{\text{e}1,2}\right)}{C^{\prime }V_{\text{CPD}}V_{\text{AC}}G\left(\omega_{\text{e}1,2}\right)}\right)}^{2}+\\ {\left(\frac{2N\left(\omega_{\text{mix}}\right)}{C^{\prime }V_{\text{AC}}^{2}G\left(\omega_{\text{mix}}\right)}\right)}^{2} \end{array}\right)}^{-1/2}$$

## E. Harmonic Mixing-KPFM (HM)

When Equation 34 is combined with ω_m_, the resulting frequency components of the electrostatic force become [10,51,83]


[50] $$\left|F_{\text{DC}}\right|=\frac{1}{2}C^{\prime }\left(V_{\text{CPD}}^{2}+\frac{1}{2}V_{\text{AC1}}^{2}+\frac{1}{2}V_{\text{AC2}}^{2}\right)$$

[51] $$\left|F_{\omega_{\text{m}}}\right|=\frac{1}{2}A_{\text{m}}C^{\prime \prime }\left(V_{\text{CPD}}^{2}+\frac{1}{2}V_{\text{AC1}}^{2}+\frac{1}{2}V_{\text{AC2}}^{2}\right)\cos\left(\omega_{\text{m}}t\right)$$

[52] $$\left|F_{\omega_{\text{e}1,2}}\right|=C^{\prime }V_{\text{CPD}}V_{\text{AC}1,2}\sin\left(\omega_{\text{e}1,2}t\right)$$

[53] $$\left|F_{2\omega_{\text{e}1,2}}\right|=\frac{1}{4}C^{\prime }V_{\text{AC}1,2}^{2}\cos\left(2\omega_{\text{e}1,2}t\right)$$

[54] $$\left|F_{\omega_{\text{m}}\pm \omega_{\text{e}1,2}}\right|=\frac{1}{2}A_{\text{m}}C^{\prime \prime }V_{\text{CPD}}V_{\text{AC}1,2}\sin\left(\left(\omega_{\text{m}}\pm \omega_{\text{e}1,2}\right)t\right)$$

[55] $$\left|F_{\omega_{\text{m}}\pm 2\omega_{\text{e}1,2}}\right|=\frac{1}{8}A_{\text{m}}C^{\prime \prime }V_{\text{AC}1,2}^{2}\cos\left(\left(\omega_{\text{m}}\pm 2\omega_{\text{e}1,2}\right)t\right)$$

[56] $$\left|F_{\omega_{\text{mix}}}\right|=\frac{1}{2}C^{\prime }V_{\text{AC}1}V_{\text{AC}2}\left(\begin{array}{l} \cos\left(\left(\omega_{\text{e}1}-\omega_{\text{e}2}\right)t\right)-\\ \cos\left(\left(\omega_{\text{e}1}+\omega_{\text{e}2}\right)t\right) \end{array}\right)$$

[57] $$\left|F_{\omega_{\text{m}}\pm \omega_{\text{mix}}}\right|=\frac{1}{4}A_{\text{m}}C^{\prime \prime }V_{\text{AC}1}V_{\text{AC}2}\left(\begin{array}{l} \sin\left(\left(\omega_{\text{e}1}+\omega_{\text{e}2}-\omega_{\text{m}}\right)t\right)\\ -\sin\left(\left(\omega_{\text{e}1}-\omega_{\text{e}2}-\omega_{\text{m}}\right)t\right)\\ +\sin\left(\left(\omega_{\text{e}1}-\omega_{\text{e}2}+\omega_{\text{m}}\right)t\right)\\ -\sin\left(\left(\omega_{\text{e}1}+\omega_{\text{e}2}+\omega_{\text{m}}\right)t\right) \end{array}\right)$$

where ω_e1_ > ω_e2_. The amplitude responses are thus


[58] $$A_{\omega_{\text{e}1,2}}=C^{\prime }V_{\text{CPD}}V_{\text{AC}1,2}G\left(\omega_{\text{e}1,2}\right)$$

[59] $$A_{2\omega_{\text{e}1,2}}=\frac{1}{4}C^{\prime }V_{\text{AC}1,2}^{2}G\left(2\omega_{\text{e}1,2}\right)$$

[60] $$A_{\omega_{\text{m}}\;\pm \;\omega_{\text{e}1,2}}=\frac{1}{2}A_{\text{m}}C^{\prime \prime }V_{\text{CPD}}V_{\text{AC}1,2}G\left(\omega_{\text{m}}\pm \omega_{\text{e}1,2}\right)$$

[61] $$A_{\omega_{\text{m}}\;\pm \;2\omega_{\text{e}1,2}}=\frac{1}{8}A_{\text{m}}C^{\prime \prime }V_{\text{AC}1,2}^{2}G\left(\omega_{\text{m}}\pm 2\omega_{\text{e}1,2}\right)$$

[62] $$A_{\omega_{\text{mix}}}=\frac{1}{2}C^{\prime }V_{\text{AC}1}V_{\text{AC}2}G\left(\omega_{\text{mix}}\right)$$

[63] $$A_{\omega_{\text{m}}\;\pm \;\omega_{\text{mix}}}=\frac{1}{4}A_{\text{m}}C^{\prime \prime }V_{\text{AC}1}V_{\text{AC}2}G\left(\omega_{\text{m}}\pm \omega_{\text{mix}}\right)$$

where ω_mix_ = ω_e1_ ± ω_e2_. Again, we will assume *V*_AC_ = *V*_AC1_ = *V*_AC2_. Thus, *V*_CPD_ may be obtained using the same approach as Equation 41 and Equation 42 using signals that are not modulated by ω_m_.

We can also obtain *V*_CPD_ using 
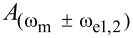
 and 
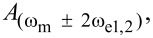
 which have a dependence on *C*″, to yield [51]


[64] $$\begin{array}{c} V_{\text{CPD(HM-DH)}}=s\frac{A_{\left(\omega_{\text{m}}\;\pm \;\omega_{\text{e}1,2}\right)}}{A_{\left(\omega_{\text{m}}\;\pm \;2\omega_{\text{e}1,2}\right)}}\cdot \\ \frac{V_{\text{AC}}}{4\text{XGain}\left(\omega_{\text{m}}\pm \omega_{\text{e}1,2},\omega_{\text{m}}\pm 2\omega_{\text{e}1,2}\right)} \end{array}$$

or by using 
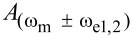
 and 
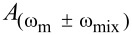
 we can obtain


[65] $$\begin{array}{c} V_{\text{CPD(HM-Mix)}}=s\frac{A_{\left(\omega_{\text{m}}\;\pm \;\omega_{\text{e}1,2}\right)}}{A_{\left(\omega_{\text{m}}\;\pm \;\omega_{\text{mix}}\right)}}\cdot \\ \frac{V_{\text{AC}}}{2\text{XGain}\left(\omega_{\text{m}}\pm \omega_{\text{e}1,2},\omega_{\text{m}}\pm \omega_{\text{mix}}\right)} \end{array}$$

We can determine the 
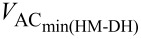
 for 
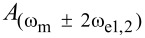
 such that


[66] $$V_{{\text{AC}}_{\text{min(HM-DH)}}}=2\sqrt{2}\sqrt{\frac{N\left(\omega_{\text{m}}\pm 2\omega_{\text{e}1,2}\right)}{A_{\text{m}}C^{\prime \prime }G\left(\omega_{\text{m}}\pm 2\omega_{\text{e}1,2}\right)}}$$

and thus


[67] $$\begin{array}{c} V_{{\text{CPD}}_{\text{min(HM-DH)}}}=\frac{N\left(\omega_{\text{m}}\pm \omega_{\text{e}1,2}\right)}{\sqrt{2}A_{\text{m}}C^{\prime \prime }G\left(\omega_{\text{m}}\pm \omega_{\text{e}1,2}\right)}\cdot \\ \frac{1}{\sqrt{\frac{N\left(\omega_{\text{m}}\pm 2\omega_{\text{e}1,2}\right)}{A_{\text{m}}C^{\prime \prime }G\left(\omega_{\text{m}}\pm 2\omega_{\text{e}1,2}\right)}}} \end{array}$$

We can determine the 
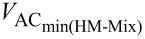
 for 
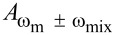
 such that


[68] $$V_{{\text{AC}}_{\text{min(HM-Mix)}}}=2\sqrt{\frac{N\left(\omega_{\text{m}}\pm \omega_{\text{mix}}\right)}{A_{\text{m}}C^{\prime \prime }G\left(\omega_{\text{m}}\pm \omega_{\text{mix}}\right)}}$$

and thus


[69] $$\begin{array}{c} V_{{\text{CPD}}_{\text{min(HM-Mix)}}}=\frac{N\left(\omega_{\text{m}}\pm \omega_{\text{e}1,2}\right)}{A_{\text{m}}C^{\prime \prime }G\left(\omega_{\text{m}}\pm \omega_{\text{e}1,2}\right)}\cdot \\ \frac{1}{\sqrt{\frac{N\left(\omega_{\text{m}}\pm \omega_{\text{mix}}\right)}{A_{\text{m}}C^{\prime \prime }G\left(\omega_{\text{m}}\pm \omega_{\text{mix}}\right)}}} \end{array}$$

Here, we note that


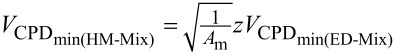


The resulting SNR is then obtained from the root sum squared of


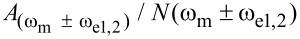


and


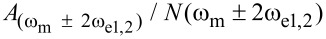


resulting in


[70] $${\text{SNR}}_{\text{HM-DH}}={\left(\begin{array}{l} {\left(\frac{2N\left(\omega_{\text{m}}\pm \omega_{\text{e}1,2}\right)}{A_{\text{m}}C^{\prime \prime }V_{\text{CPD}}V_{\text{AC}}G\left(\omega_{\text{m}}\pm \omega_{\text{e}1,2}\right)}\right)}^{2}+\\ {\left(\frac{8N\left(\omega_{\text{m}}\pm 2\omega_{\text{e}1,2}\right)}{A_{\text{m}}C^{\prime \prime }V_{\text{AC}}^{2}G\left(\omega_{\text{m}}\pm 2\omega_{\text{e}1,2}\right)}\right)}^{2} \end{array}\right)}^{-1/2}$$

Similarly, when using the root sum squared of


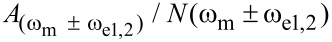


and


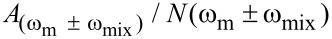


we obtain


[71] $${\text{SNR}}_{\text{HM-Mix}}={\left(\begin{array}{l} {\left(\frac{2N\left(\omega_{\text{m}}\pm \omega_{\text{e}1,2}\right)}{A_{\text{m}}C^{\prime \prime }V_{\text{CPD}}V_{\text{AC}}G\left(\omega_{\text{m}}\pm \omega_{\text{e}1,2}\right)}\right)}^{2}+\\ {\left(\frac{4N\left(\omega_{\text{m}}\pm \omega_{\text{mix}}\right)}{A_{\text{m}}C^{\prime \prime }V_{\text{AC}}^{2}G\left(\omega_{\text{m}}\pm \omega_{\text{mix}}\right)}\right)}^{2} \end{array}\right)}^{-1/2}$$

## Appendix III: Summary of Drive and Detection Schemes

For a summary of drive and detection schemes see Table 3.

**Table 3:** Summary of drive and detection (Det.) schemes.^a^

Mode	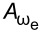	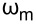	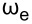 Drive	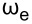 Det.	*V*_CPD_ ∝	*C*′/*C*″	*V*_AC_ ∝	Advantages	Disadvantages

AM^b^	ω_1_	ω_2_^c^	ω_1_	ω_1_	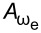	*C*′	*Q*_1_/*k*_1_	widely available high sensitivity	low spatial resolution
	ω_2_	ω_1_^c^	ω_2_	ω_2_	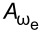	*C*′	*Q*_2_/*k*_2_		

DH	ω_1_	ω_2_^c^	ω_1_	ω_1_ 2ω_1_	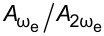	*C*′	*Q*_1_/*k*_1_	single pass scan compatible	low spatial resolution frequency placement poor SNR reliance on XGain
	ω_2_	ω_1_^c^	ω_2_	ω_2_ 2ω_2_	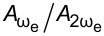	*C*′	*Q*_2_/*k*_2_		

ED-DH	ω_1_	–	ω_1_ ω_2_/2	ω_1_ ω_2_	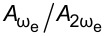	*C*′	*Q*_1_/*k*_1_	flexible frequency placement high bandwidth	low spatial resolution difficult to use in single pass reliance on XGain
	ω_2_	–	ω_2_ ω_1_/2	ω_2_ ω_1_	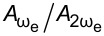	*C*′	*Q*_2_/*k*_2_		

ED-Mix	ω_1_	–	ω_1_ (ω_2_ ± ω_1_)	ω_1_ ω_2_	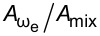	*C*′	*Q*_1_/*k*_1_	sensitivity > ED-DH flexible frequency placement high bandwidth	low spatial resolution difficult to use in single pass reliance on XGain
	ω_2_	–	ω_2_ (ω_2_ ± ω_1_)	ω_2_ ω_1_	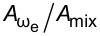	*C*′	*Q*_2_/*k*_2_		

Het^b^	ω_1_	ω_2_	(ω_2_ ± ω_1_)	ω_1_	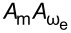	*C*″	*A*_m_*Q*_1_/*k*_1_	sensitivity > FM-KPFM high bandwidth high spatial resolution	sensitivity < AM higher bias required
	ω_2_	ω_1_	(ω_2_ ± ω_1_)	ω_2_	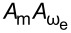	*C*″	*A*_m_*Q*_2_/*k*_2_		

Het-DH	ω_1_	ω_2_	(ω_2_ ± ω_1_)	ω_1_ 2ω_1_	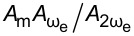	*C*″	*A*_m_*Q*_1_/*k*_1_	high bandwidth high spatial resolution	sensitivity < DH frequency placement reliance on XGain
	ω_2_	ω_1_	(ω_2_ ± ω_1_)	ω_2_ 1ω_2_	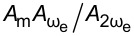	*C*″	*A*_m_*Q*_2_/*k*_2_		

HM-DH	ω_1_	ω_n_	(ω_n_ ± ω_1_) (ω_n_ ± ω_2_/2)	ω_1_ ω_2_	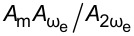	*C*″	*A*_m_*Q*_1_/*k*_1_	flexible frequency placement high bandwidth high spatial resolution	sensitivity < ED-DH frequency placement reliance on XGain
	ω_2_	ω_n_	(ω_n_ ± ω_2_) (ω_n_ ± ω_1_/2)	ω_2_ ω_1_	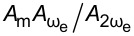	*C*″	*A*_m_*Q*_2_/*k*_2_		

HM-Mix	ω_1_	ω_n_	(ω_n_ ± ω_1_) (ω_n_ ± ω_2_)	ω_1_ ω_2_	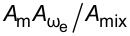	*C*″	*A*_m_*Q*_1_/*k*_1_	flexible frequency placement high bandwidth high spatial resolution	sensitivity < ED-Mix reliance on XGain
	ω_2_	ω_n_	(ω_n_ ± ω_2_) (ω_n_ ± ω_1_)	ω_2_ ω_1_	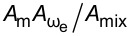	*C*″	*A*_m_*Q*_2_/*k*_2_		

^a^ω*_n_* = mechanical oscillation, on another eigenmode or off resonance, at a frequency which does not interfere with electrical drive or detection and results in an oscillation amplitude, *A*_m_. Off-resonance conditions imply all electrical detection frequencies ω → 0 Hz. ^b^Techniques that would typically be operated in a closed loop configuration. ^c^The drive is optional.
